# Annotations

**Published:** 1891-11-28

**Authors:** 


### ANNOTATIONS.

**More Light upon Inoculation.**

THE Waynflete Professor of Physiology at Oxford, Dr. Burdon Sanderson, F.R.S., in his Croonian Lectures delivered before the Royal College of Physicians, has expounded certain inoculation principles with much logical clearness, and with beautiful simplicity. Those who are interested in the subject of inoculation and vaccination—that is, all really intelligent persons—should read and think over Professor Sanderson's lectures. Most readers are familiar with the idea of first attenuating a given virus; then inoculating with it a subject to be protected, and a resulting immunity in the subject. Dr. Burdon Sanderson has, so to speak, expounded to us the philosophy of these processes. It seems to be established that the disease-producing power of a particular germ is in direct proportion to the degree of its attenuation. The more attenuated is a given virus, the less is its power of giving rise to its specific morbid results. Dr. Burdon Sanderson has not shown us whether the protection produced by inoculation with a weak virus is of the same trustworthy character as that brought about by the action of a stronger virus. *A priori*, one would suppose that the resulting protection must be directly proportionate to the severity of the morbid process, as that in its turn is proportionate to the energy of the virus. There are plenty of facts bearing upon this point; but, so far as we know, they have not yet been dealt with in any scientific way. Many people are under the impression that the chief and practically the only method of attenuating a virus is that of cultivation as practised by Pasteur. But Professor Sanderson reminds us that oxygen, pure air, is a powerful attenuator. Certain micro-organisms cannot live in pure oxygen at all. To destroy them, it is sufficient to expose them freely to the atmosphere. Certain other organisms, as analogy has taught us to expect, thrive well in oxygen. But these, when very abundantly supplied with this element, develop with abnormal rapidity, and thus attenuate themselves by the too great energy of their growth. In both these cases atmospheric air is an attenuator, and therefore a protector. A third method of attenuation is the chemical. This consists of adding to the virus a substance which, though not strong enough to destroy it, is yet sufficient to impair its growth. Among chemical attenuators what are known as disinfectants have a high place. One substance, bi-chromate of potash, is said to yield an attenuated virus as weak and as permanent as either of the other methods employed. The important facts to be noted are that what are called vaccines are numerous; that they can be perfectly controlled as to their morbific potency by at least three easily managed methods; and that, when properly used, such vaccines seem to produce the results of more or less decided immunity against certain diseases. It will be seen that we do not commit ourselves to any very emphatic judgments. But we do desire to keep all classes of readers alive to the fact that investigators of the most competent and experienced order are making researches into every department of the great inoculation question. The public therefore, as well as the medical profession, should keep an open mind. The questions at issue cannot be settled by popular newspaper controversy, but only by patient and repeated experiment extending over a large area, and perhaps continuing for a great number of years.

**Where should Hospitals be Placed?**

LONDON is steadily becoming more and more unsuitable as a place for the treatment of sick persons who are suffering from idiopathic diseases as distinguished from accidental injuries. It is admitted that physicians of the most conspicuous ability practise in London; but even London physicians cannot cure without the proper means of cure, any more than an army can fight without weapons or without a commissariat. Next to food the most important of all the conditions of health is pure air. This is true even for those who are well. They cannot keep well for any length of time without a supply of pure and wholesome air in adequate quantity. But if it be true for those who are well and who can spend a great deal of time every day out of doors, how much more true is it for those who are sick and who have to live day and night within closed doors, and in the company of scores or hundreds of other persons who are sick like themselves. For quite six months in the year London is one of the unfittest places in the world for sick people. Its crowds, its poisonous river, its smoke, its fog, make it a place dismal, unwholesome, and melancholy beyond compare. Hospitals for the chronic sick should be built at a high elevation, on gravel or sandy soil, and in the midst of the purest air that the fresh country hills can supply. Not a single distinguished physician in London would deny this if he were anything but a London physician. He will probably contend that the superior skill of the metropolitan practitioner counterbalances the want of fresh air. But such a contention is not true; it is very far from the truth—a second or third-rate physician in a pure atmosphere will work far more and better cures than a first-rate physician surrounded by impure air. Hospitals, we shall be told, must be retained in London for the sake of their medical schools. This is not at all necessary. The country is a much fitter place for medical study, if plenty of patients are taken there, than the town. We shall no doubt always require "accident" hospitals in London, and in London and other large towns that department of medicine called surgery will still have to be mainly studied. But general medicine can be studied wherever patients can be gathered together; and the country offers so many advantages to patients and students alike, that it must ultimately become the seat of all general medical schools. What about the teachers? Says the metropolitan advocate: Where will you get your eminent physicians to teach your students? We do not want eminent physicians to teach students—we want eminent teachers. The eminent physician is often a most miserable teacher; and that for the very sufficient reason that teaching is an accident of his career and not the essence of his work. If medicine is to take its proper place alongside the other learned professions it must develop a class of teachers whose bread and name and fame must depend entirely upon their success in teaching. It is plain that the present system of crowding hospitals together in London, and other such abodes of innumerable atmospheric poisons, is not only ruinous to patients, but injurious to students and to medical science generally. We shall have to face the inevitable, and to remove the great hospitals and medical schools to the country sooner or later; and that being so, the sooner we do it the better for all parties concerned.

**The Scheduling of Poisons.**

A CORRESPONDENT sends us an account of the suicide of a young woman at Norwich by means of carbolic acid. The girl, for she was only twenty-three years of age, left a letter for her relatives, in which she said, "I have taken carbolic acid. I purchased it myself this morning." According to the account given, the young woman complained of feeling unwell about three o'clock in the afternoon. A medical man was sent for, who ordered her to bed, and remained with her until her death, which took place about five o'clock. At the inquest the medical man stated that he had seen a pewter pot after the suicide's death which contained what appeared to be a mixture of carbolic acid and stout. A teaspoonful of carbolic acid was sufficient to kill any person. Carbolic acid was a poison not scheduled in the Pharmacy Act. The obvious conclusion on reading a story of this kind is that carbolic acid ought to be scheduled as a poison in the Pharmacy Act. This young woman was but twenty-three, and so far as the evidence given at the inquest showed, had really no very urgent reason for wishing to take away her life. If she had found it impossible to obtain a poison readily, she might have consulted a medical man, and been restored to health in two or three weeks. We cannot contemplate an increase of suicides with anything but grave concern. In a population of thirty-five millions, one death more or less is, in a sense, a matter of no great moment. But the temper of mind which prompts a person in a fit of disappointment, or when labouring under more serious trouble, to put an end to her own life, is a temper which if widely prevalent threatens serious injury to all those that are left to bear the social and family burdens which the suicide so readily throws down. It is a ridiculous thing to schedule any poisons at all unless we schedule all substances that are actively and promptly poisonous. Let us be consistent. Either make suicide as easy as possible, or as difficult as possible—one of the two. But do not let us, with ludicrous inconsequence, put certain poisons absolutely beyond the reach of the public, and leave others, equally dangerous, to be bought by the first man who can spare a penny or twopence for the purchase.

